# Co-Occurrence of NDM-5 and RmtB in a Clinical Isolate of *Escherichia coli* Belonging to CC354 in Latin America

**DOI:** 10.3389/fcimb.2021.654852

**Published:** 2021-04-29

**Authors:** Agustina Costa, Roque Figueroa-Espinosa, Florencia Gaudenzi, Nilton Lincopan, Bruna Fuga, Barbara Ghiglione, Gabriel Gutkind, José Di Conza

**Affiliations:** ^1^ Laboratorio de Resistencia Bacteriana, Instituto de Bacteriología y Virología Molecular (IBaViM), Facultad de Farmacia y Bioquímica, Universidad de Buenos Aires, Ciudad Autónoma de Buenos Aires, Argentina; ^2^ Consejo Nacional de Investigaciones Científicas y Técnicas, Ciudad Autónoma de Buenos Aires, Argentina; ^3^ Laboratorio de Bacteriología, Hospital Central de San Isidro “Dr. Melchor Ángel Posse, ”, Martínez, Argentina; ^4^ Departamento de Microbiologia, Instituto de Ciências Biomédicas, Universidade de São Paulo, São Paulo, Brazil

**Keywords:** metallo-β-lactamase, NDM-5, RmtB, *Escherichia coli*, antibiotic multi-resistance

## Abstract

New Delhi metallo-β-lactamase (NDM)-producing isolates are usually resistant to most β-lactams and other antibiotics as a result of the coexistence of several resistance markers, and they cause a variety of infections associated to high mortality rates. Although NDM-1 is the most prevalent one, other variants are increasing their frequency worldwide. In this study we describe the first clinical isolate of NDM-5- and RmtB-producing *Escherichia coli* in Latin America. *E. coli* (Ec265) was recovered from a urine sample of a female outpatient. Phenotypical and genotypical characterization of resistance markers and conjugation assays were performed. Genetic analysis of Ec265 was achieved by whole genome sequencing. Ec265 belonging to ST9693 (CC354), displayed resistance to most β-lactams (including carbapenems), aminoglycosides (gentamicin and amikacin), and quinolones. Several resistance genes were found, including *bla*_NDM-5_ and *rmtB*, located on a conjugative plasmid. *bla*_NDM-5_ genetic context is similar to others found around the world. Co-transfer of multiple antimicrobial resistance genes represents a particular challenge for treatment in clinical settings, whereas the spread of pathogens resistant to last resort antibiotics should raise an alarm in the healthcare system worldwide.

## Introduction

NDM metallo-β-lactamases are carbapenemases capable of hydrolyzing almost all β-lactam antibiotics (except aztreonam), being found in several species of *Enterobacterales, Acinetobacter*, and *Pseudomonas*. NDM-producing isolates are usually resistant to most antibiotics due to coexistence of several resistance determinants, and they cause a variety of infections associated with high mortality rates (Mojica et al., 2016). Even though 31 NDM variants have been already described (https://www.bldb.eu, accessed 04.06.21), the NDM-1 variant remains by far the most prevalent worldwide. Substitutions have been observed at 25 of the 270 amino acid positions, of which M154L is present in 11 out of all the distinct variants (Basu, 2020). In contrast to NDM-1 that is widely spread among several *Enterobacterales* and other Gram-negative bacilli, NDM-5 seems to be more restricted to *Escherichia coli* isolates (Wu et al., 2019), as it was the first to be reported from a patient in the United Kingdom after previous hospitalization in India (Hornsey et al., 2011). In the Americas, NDM-5 has been only described in clinical isolates in the USA (Mediavilla et al., 2016; Flerlage et al., 2020) and, to the best of our knowledge; it has not been reported in human clinical samples in Latin America, so far. We are aware that the emergence of a novel variant in our geographical region could lead to the replacement or substitution of the prevailing carbapenemases present in the area. On the other hand, RmtB is a 16S ribosomal RNA methyltransferase conferring high-level resistance to aminoglycosides, which has already been disseminated among *Enterobacterales* worldwide (Doi et al., 2016). This situation has become worrisome due to the loss of one of the most important therapeutic options for treatment of severe infections caused by carbapenem-resistant *Enterobacterales* (CRE), including those produced by NDM-positive pathogens.

The aim of this study is to report the first clinical isolate of NDM-5- and RmtB-producing *E. coli* in Latin America and to describe the genetic context of these genes.

## Material and Methods

On September 2018, a female outpatient was admitted to Hospital Central de San Isidro “Dr Melchor Ángel Posse” (Buenos Aires Province, Argentina), presenting urinary tract infection symptoms. The patient lived in a retirement nursing home and had not traveled abroad during the prior 12 months. No information about previous antibiotic treatment was recorded at sampling time, nor in the hospital records. A carbapenem-resistant *E. coli* isolate (Ec265) was recovered from a urine sample. The patient was successfully treated with nitrofurantoin and hydration.

Bacterial identification was carried out by matrix assisted desorption-ionization time of flight mass spectrometry (MALDI-TOF MS) (Bruker Daltonics, Germany).

Susceptibility testing was performed by disk diffusion test according to the Clinical Laboratory Standards Institute (CLSI) recommendations (CLSI, 2020). Phenotypic screening of metallo-β-lactamases (MBL) was performed by synergy tests using meropenem (10 µg)-, EDTA (1 µmol)-, and imipenem (10 µg)-containing disks. Minimal Inhibitory Concentrations (MIC) were determined by Sensititre ARGNF Kit (Thermo Scientific). Results were interpreted according to CLSI guidelines (CLSI, 2020), except colistin and tigecycline for which the EUCAST 2020 breakpoints were considered (https://www.eucast.org, accessed 04.06.21).

Detection and characterization of NDM genes were performed by polymerase chain reaction (PCR), cloning, and sequencing. For *bla*_NDM_ detection, NDM-F (5´-GGTTTGGCGATCTGGTTTTC-3´) and NDM-R (5´-CGGAATGGCTCATCACGATC-3´) primers were used, rendering a 621 bp product. For *bla*_NDM_ cloning into a pK19 vector, custom primers including restriction sites were designed (NDM-SalF 5´- TACGCGTCGACATGGAATTGCCCAAT-3´ and NDM-EcoR 5´- CGGAATTCTCAGCGCAGCTTGTC-3´), and *E. coli* TOP10 was used as the recipient strain, which was transformed with the recombinant construction. Recombinants were selected on Tryptic Soy Agar containing 30 µg/ml kanamycin and 4 µg/ml meropenem, and checked by disk diffusion tests, followed by *bla*_NDM_ amplification using M13_pUC forward (5′-CCCAGTCACGACGTTGTAAAACG-3´) and M13_pUC reverse (5´-CAGGAAACAGCTATGAC-3´) primers.

Other β-lactamase genes such as *bla*_CTX-M_-type and frequent *bla*_OXA_-variants were screened by PCR, and detection of the *rmtB* gene was also performed by PCR (RmtB-F 5´-ACTTTTACAATCCCTCAATAC-3´ and RmtB-R 5´-AAGTATATAAGTTCTGTTCCG-3´) (Berçot et al., 2011).

Phylogenetic group was carried out by PCR following the Clermont scheme (Clermont et al., 2013).

Plasmid conjugation was performed by a mating-out assay using *E. coli* J53 (sodium azide resistant) as recipient, and Luria Bertani agar plates supplemented with sodium azide (250 µg/ml) and cefotaxime (2 µg/ml) as selective agents. A REP-ERIC PCR assay was carried out to evaluate the relationship between transconjugant and recipient strains (Versalovic et al., 1991).

Finally, whole genome sequencing (WGS) of the clinical isolate Ec265 was carried out on the Illumina NextSeq platform (San Diego, USA) using a paired-end (PE) library. Briefly, single colonies of the bacteria were grown in 3 ml of lysogeny broth for 18 h at 37°C and the DNA was extracted using a PureLink quick gel extraction kit (Life Technologies, CA, USA). The total genomic DNA was used to library construction with a Nextera DNA Flex kit (Illumina, San Diego, CA, USA). The generated raw reads were initially subjected to quality check using FastQC software (https://www.bioinformatics.babraham.ac.uk/projects/fastqc, accessed 01.17.21), and the paired-reads trimmed to remove adapters and low-quality regions (PHRED quality score below 20) using TrimGalore v0.6.5 (https://github.com/FelixKrueger/TrimGalore, accessed 01.17.21).

Sequence reads were assembled *de novo* using SPAdes V3.9, and analyzed by using on-line bioinformatic tools (Center for genomic epidemiology, CARD and Pathogen watch). Multilocus sequence typing (MLST) was determined following Achtman and Pasteur schemes (https://pubmlst.org/escherichia/, accessed 01.17.21). EnteroBase (https://enterobase.warwick.ac.uk/, accessed April, 2021) was used to determine core genetic relationships among globally disseminated *E. coli* strains with identical sequence type, as well as to define the clonal complex (CC). To this end, the Ec265 strain was analyzed considering single locus variants (SNV) and double locus variants (DLV). Reads were submitted to EnteroBase under accession number ESC_OA7444AA.

## Results

### Identification and Susceptibility Profile

Ec265 was identified as *Escherichia coli* (score value ≥2.0, indicating a reliable identification at specie level), displaying a multidrug-resistant profile to penicillins (ampicillin), cephalosporins (cephalotin, cefuroxime, cefoxitin, cefotaxime, ceftazidime, and cefepime), carbapenems (imipenem, meropenem, and ertapenem), aminoglycosides (gentamycin and amikacin), trimethoprim-sulfamethoxazole, tetracycline, and fluoroquinolones (ciprofloxacin and levofloxacin); remaining susceptible to colistin, nitrofurantoin, fosfomycin, tigecycline, and aztreonam. Synergy was observed between EDTA and both meropenem and imipenem disks, suggesting MBL presence. MIC values are shown on **Table 1**.

**Table 1:** MIC values for Ec265, transconjugant, and recipient strains.

Antibiotic	MIC (µg/ml)		
	Ec265	Tc265	*E. coli* J53
Ampicillin	>16	>16	≤8
Ampicillin/sulbactam	>16/8	>16/8	≤8/4
Amoxicillin/clavulanic acid	>16/8	>16/8	≤8/4
Cephalotin	>32	>32	16
Cefuroxime	>16	>16	≤4
Cefoxitin	>16	>16	≤8
Cefotaxime	>32	>32	≤1
Ceftazidime	>32	>32	≤2
Cefepime	>16	>16	≤2
Aztreonam	≤8	≤8	≤8
Imipenem	8	8	≤1
Meropenem	>16	16	≤1
Ertapenem	>2	>2	≤1
Gentamicin	>8	>8	≤4
Amikacin	>32	>32	≤8
Nalidixic acid	>16	≤16	≤16
Ciprofloxacin	>2	0.12	0.12
Levofloxacin	>4	0.5	0.5
Nitrofurantoin	≤32	≤32	≤32
Fosfomycin	≤32	≤32	≤32
Chloramphenicol	16	16	<8
Trimethoprim/sulfamethoxazole	>2/38	>2/38	≤2/38
Colistin	≤1	≤1	≤1

### Resistance Markers and Mobilization

The *E. coli* strain Ec265, belonging to phylogenetic group F, was positive for *bla*_NDM_ gene detection. PCR for *bla*_CTX-M_ and *bla*_OXA_ genes rendered negative results. The *bla*_NDM_ gene was successfully transferred to *E. coli* J53 showing that this resistance marker was located on a conjugative plasmid. Resistance to gentamicin and amikacin was co-transferred in the conjugation assay (**Table 1**). Presence of the *rmtB* gene was detected in both Ec265 and the transconjugant (Tc265). REP-ERIC PCR discarded any clonal relationship between them. Recombinant pK19 plasmid harboring the *bla*_NDM_ gene was sequenced and the *bla*_NDM-5_ gene was confirmed. Recombinant strain also displayed a resistant phenotype against meropenem (8 µg/ml) and imipenem (>16 µg/ml).

Because of the unusual variant of *bla*_NDM_ for our region, and the resulting resistance in the clinical isolate, a deep genomic analysis of Ec265 was performed by WGS.

### Whole Genome Sequencing

WGS analysis revealed a 5 171 045-bp genome size, with 50.5% GC content, 166 contigs (>200 bp), and N50 value of 162 218 bp (GenBank accession no. **JACXXI000000000**). Resistome analysis of Ec265 predicted several acquired antimicrobial resistance genes, such as *rmtB*, *aac(3)-IId*, *aadA2*, *aph(3´´)-Ib*, *aph(6)-Id* (aminoglycoside resistance), *dfrA12* and *dfrA17* (trimethoprim resistance), *bla*_NDM-5_ and *bla*_TEM-1B_ (β-lactam resistance), *sul1* and *sul2* (sulfonamide resistance), *erm(B)* and *mph(A)* (macrolide resistance), *tet(B*) (tetracycline resistance) and chromosomal point mutations in *parE* (I355T), *parC* (S80I, E84G), and *gyrA* (S83L, D87N), involved in fluoroquinolones resistance (**Table 2**).

**Table 2:** Genomic characteristics of Ec265 isolate.

Characteristics	*Escherichia coli* Ec265
Genome data	
Genome size (bp)	5 171 045
% GC content	50,5
N50 (bp)	162 218
Resistome	
Antibiotics	
β-lactams	*bla*_NDM-5_, *bla*_TEM-1B_
Aminoglycosides	*rmtB*, *aac(3)-IId*, *aadA2*, *aph(3´´)-Ib*, *aph(6)-Id*
Trimethoprim	*dfrA12*, *dfrA17*
Sulfonamides	*sul1*, *sul2*
Macrolides	*erm(B)*, *mph(A)*
Tetracycline	*tet(B*)
Chromosomal point mutations	
Fluoroquinolones	*parE* (I355T), *parC* (S80I, E84G), *gyrA* (S83L, D87N)
Acquired virulence factors	*air*, *eilA*, *gad*, *lpfA*, *mcmA*
Plasmids	IncFIA, IncFIB, IncFII, IncQ1, Col, and Col156
GenBank accession number	JACXXI000000000

Ec265 belongs to Fim typeH58 CH type 88-58 (*fimH58* and *fumC88*), and the serotype predicted was O153:H34. Some acquired virulence factors were identified, such as *air* (enteroaggregative immunoglobulin repeat protein), *eilA* (*Salmonella* HilA homolog), *gad* (glutamate decarboxylase), *lpfA* (long polar fimbriae), and *mcmA* (microcin M part of colicin H) (**Table 2**).

According to PlasmidFinder (https://cge.cbs.dtu.dk/services/PlasmidFinder/, accessed 01.17.21), Ec265 harbors different replicon types: IncFIA, IncFIB, IncFII, and IncQ1; however, when Pathogenwatch software (https://pathogen.watch/, accessed 01.17.21) was used, Col and Col156 replicon types were also detected (**Table 2**). There are several replicon types reported to be associated with *bla*_NDM-5_ carrying plasmids in *Enterobacterales* worldwide, being IncX3 the most common, followed by IncFIB, IncFII, and IncFIA (Wu et al., 2019). Two of these frequent replicons were detected in Ec265 (IncFII and IncFIB). The potential location of *bla*_NDM-5_ in an IncFII-type plasmid is of concern considering its known capacity of efficient spread among bacteria (Bonnin et al., 2012).

**Figure 1:**
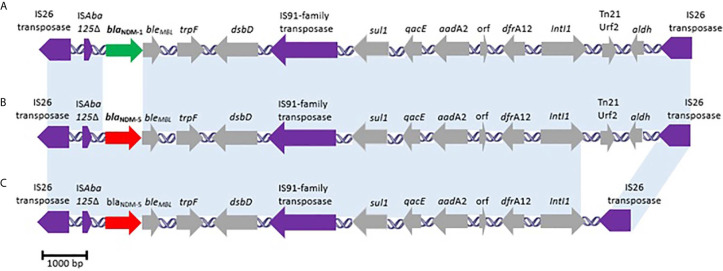
Schematic representation of *bla*_NDM_ contexts of Ec265 and other *E*. *coli* human isolates worldwide disseminated: **(A)** GenBank accession no. **JQ364967** (skin, France); **(B)** Ec265 strain isolated from urine, in Argentina (GenBank accession no. **JACXXI000000000**; **(C)** the same sequence correspond to three clinical strains isolated from rectal swab in Canada (GenBank accession no. **CP023871**), blood in Myanmar (GenBank accession no. **AP018147**), and urine in Italy (GenBank accession no. **MN007141**). The light-blue bands indicate identical gene arrangement.

The contig in which *bla*_NDM-5_ was found according to the assembly (contig 54, 10 926 bp) was annotated with RAST software in order to determine its genetic context. A truncated insertion sequence IS*Aba*125 was found upstream of *bla*_NDM-5_, whereas bleomycin resistance gene (*ble*_MBL_), that encodes a bleomycin resistance protein (BRP), was observed downstream (**Figure 1B**). These two features are shared with other common genetic contexts of *bla*_NDM_ (Wu et al., 2019). Some of the elements of NDM-5 genetic context (IS*Aba125* truncated sequence, IS*91*-family transposase, and Tn*21* Urf2) code for transposase products, that may have had a role in the mobilization of this gene from other genetic platforms. Genes encoding BRP and NDM are co-expressed from the same promoter (Dortet et al., 2017), and could be co-selected either by bleomycin-like molecules (cancer treatment drugs) or carbapenems. Further downstream of *ble*_MBL_, there are a set of several genes, including *trp*F (encoding a phosphoribosyl anthranilate isomerase), *dsb*D (encoding a protein disulfide reductase), and a typical class 1 Integron containing *sul1*, *qacE*, *aadA2*, and *dfrA12* resistance genes. Finally, the genetic region containing *bla*_NDM-5_ and class 1 Integron is flanked by two copies of IS*26* transposase that delimit a small resistance island.

Ec265 *bla*_NDM-5_ context is the same as the one observed in a clinical strain of *E. coli* from France containing *bla*_NDM-1_ (**Figures 1A, B**). Furthermore, considering the *bla*_NDM-5_ contexts of greater identity corresponding to clinical strains from other countries around the world (Canada, Myanmar, and Italy), they did not show the presence of Urf2 and *aldh* elements (**Figures 1B, C**). All these strains mentioned above contain IncFII-type plasmids harboring *bla*_NDM-1_ or *bla*_NDM-5_.

Two genes codifying a proton antiporter (*cdu*2) and the chaperonin GroEL were located downstream of *rmtB* (contig 87). This 1.68 kb-arrangement was 100% identical to other *rmtB-*containing *Enterobacterales* (GenBank accession nos. **CP050367**, **MN061455**, **MN007141**) recovered from clinical samples.

According to Achtman scheme, Ec265 belongs to ST9693 (allelic profile: *adk*-85, *fumC*-88, *gyrB*-78, *icd*-29, *mdh*-59, *purA*-5, *recA*-62) and according to Pasteur database, it belongs to ST39 (allelic profile: *dinB*-13, *icdA*-39, *pabB*-11, *polB*-16, *putP*-12, *trpA*-25, *trpB*-8, *uidA*-19). Ec265 reported in this study do not belong to STs commonly associated to *E. coli* NDM-producing isolates elsewhere (such as ST101, ST167, ST131, ST405, ST410, and ST648) (Dadashi et al., 2019). EnteroBase software, which works with Achtman scheme (7 MLST genes), showed that ST9693 belongs to CC354 and to date, there was only one more *E. coli* ST9693 isolate record from Norway (April 2021). The analysis of closely related STs, such as SLVs, displayed a total of 477 *E. coli* isolates, of which 457 (96%) belonged to the international ST354. The inclusion of DLVs rendered 31 more isolates (a total of 508), grouped in 29 different STs belonging to CC354.

ST354 has been previously reported in carbapenem-resistant *E. coli* clinical isolates harboring NDM-5 (Zhang et al., 2016; Aung et al., 2018) and KPC (Zouh et al., 2018) in Asian countries meanwhile only in KPC-producing *E. coli* ST2287, a SLV of ST354 complex, in USA (Chavda et al., 2016). Although ST354 has been associated with *E. coli* isolates resistant to several antibiotics, recovered from humans (Dadashi et al., 2019) and animals (Guo et al., 2015; Zhuge et al., 2020), it is not considered a high-risk clone.

## Discussion

To our best knowledge, even if NDM-5-producing clinical isolates have been reported in other countries around the world, so far in Latin America, essentially *bla*_NDM-1_ has been reported (Wu et al., 2019), while *bla*_NDM-5_ was only described in Brazil in an *Enterobacter bugandensis* isolate from an environmental sample (Matteoli et al., 2020). This work would be the first report of a clinical isolate of carbapenem-resistant *E. coli* carrying NDM-5 in our country and region, that also displays resistance to amikacin and fluoroquinolones. It is important to highlight that previously, the replacement of ESBLs in Argentina (CTX-M-2 by CTX-M-15 hegemony shift) happened in an unnoticed way until detected by searching for specific resistance markers instead of general resistance mechanisms (Sennati et al., 2012) showing the significance of reporting novel variants to understand the changing epidemiology of countries and regions. Besides, we demonstrated *bla*_NDM-5_ localization in a conjugative plasmid, raising an alert about the potential dissemination of this resistance marker to a high-risk clone, in addition to the fact that this NDM variant display higher levels of resistance when compared to NDM-1 (Hornsey et al., 2011).

The co-transfer of multiple antimicrobial resistance genes represents a particular challenge for clinical treatment in health care settings, and the spread of isolates resistant to last resort antibiotics, such as carbapenems and amikacin, should be a global warning in public health that deserves close monitoring.

## Data Availability Statement

The datasets presented in this study can be found in online repositories. The names of the repository/repositories and accession number(s) can be found below: https://www.ncbi.nlm.nih.gov/genbank/, JACXXI000000000.

## Author Contributions

GG and JDC conceived and designed the experiments. AC, BG, RF-E, GG, and JDC wrote the manuscript. AC, RF-E, and JDC analyzed the data. FG supplied the clinical isolate and performed the phenotypical characterization. AC performed the experiments. NL and BF performed WGS. All authors contributed to the article and approved the submitted version.

## Funding

This work was supported by Agencia Nacional de Promoción Científica y Tecnológica PICT 2018-3189 to BG, PICT 2015-1925 and UBACyT 2018 - 20020170100473BA to GG and Conselho Nacional de Desenvolvimento Científico e Tecnológico (grants AMR 443819/2018-1, 433128/2018-6, and 312249/2017-9) to NL.

## Conflict of Interest

The authors declare that the research was conducted in the absence of any commercial or financial relationships that could be construed as a potential conflict of interest.
